# Special Issue: G Protein-Coupled Adenosine Receptors: Molecular Aspects and Beyond

**DOI:** 10.3390/ijms21061997

**Published:** 2020-03-15

**Authors:** Francisco Ciruela

**Affiliations:** 1 Unitat de Farmacologia, Departament de Patologia i Terapèutica Experimental, Facultat de Medicina i Ciències de la Salut, IDIBELL, Universitat de Barcelona, 08907 L’Hospitalet de Llobregat, Spain; fciruela@ub.edu; 2 Institut de Neurociències, Universitat de Barcelona, 08035 Barcelona, Spain

Adenosine is a purine nucleoside present in all human cells where it plays many different physiological roles: From being a building block for nucleic acids to a key constituent of the biological energy currency ATP [1]. Indeed, more than 90 years ago, Drury and Szent-Györgyi reported that adenosine produces profound hypotension and bradycardia [2], and until the present time, the list of physiological effects of adenosine has expanded considerably [3]. In addition, adenosine is a well-known neuromodulator in the brain and has effects on other tissues, thus exerting its physiological actions through four different subtypes of G protein-coupled adenosine receptors (i.e., A_1_R, A_2A_R, A_2B_R and A_3_R) which, as expected, are expressed in a large variety of cells throughout the body [4]. Consequently, ARs are potential therapeutic targets in a variety of pathophysiological conditions, including cancer, cardiovascular diseases, neurological disorders, and inflammatory and autoimmune diseases [5].

Consequently, the interest in the molecular structure and pharmacology of ARs has increased in recent years. Interestingly, more than 30 crystal structures for human ARs have been reported in the last decade, thus, they are the most structurally characterized G protein-coupled receptor at the molecular level. In addition, selective agonists and antagonists for all four AR subtypes have been developed and their diagnostic and therapeutic utility is being pursued.

The following special issue of the International Journal of Molecular Sciences aims at providing the recent developments in ARs from several points of view, thus going from mechanistic aspects of ligand-receptor interaction to physiopathological features involving adenosine receptors. Accordingly, four review articles highlight the relevance of AR targeting in some pathologies and the pharmacotherapeutic usefulness of targeting these receptors. Fenouillet et al. review the concept of “receptor reserve”, also known as “spare receptors”, in the field of adenosinergic transmission and its implication in cardiovascular disorders [6]. Interestingly, AR reserve allows adenosine to achieve its maximal efficacy without the need of occupying all cell ARs. As indicated by the authors [6], spare ARs within the cardiovascular system appear to compensate for a low extracellular adenosine level and/or a low adenosine receptor number, such as in coronary artery disease (CAD) [7] or some kinds of neurocardiogenic syncope [8]. Thus, the existence of spare receptors appears to be an attempt to overcome a weak interaction between adenosine and its receptors. Finally, the authors hypothesize that the identification of adenosine spare receptors in cardiovascular disorders may be helpful for diagnostic purposes. Next, Wolska and Rozalski further review the current knowledge of using synthetic, selective, longer-lasting agonists for A_2A_R and A_2B_R on platelet function inhibition, thus assessing their potential use for anti-platelet pharmacotherapy [9]. Hence, the authors highlight the renewed interest in using A_2A_R agonists as anti-platelet therapy in the management of arterial thrombosis, a disorder that often results in cardiovascular disease and stroke. Interestingly, the combination in a multimodal fashion of A_2A_R agonists (i.e., NECA, HE-NECA, CGS 21680, 2-chloroadenosine and PSB-15826) with other purinergic-based anti-platelet agents, for instance P2Y_12_ receptor antagonist (i.e., cangrelor, clopidogrel or prasugrel), may represent a promising approach to prevent thrombotic events [9]. Gao and Jacobson discuss the role of A_2B_R in cancer [10]. Interestingly, while all four ARs are reported to be somehow involved in cancer progression [11], A_2B_R signaling constitutes a major pathway contributing to cancer cell proliferation and solid tumor growth, angiogenesis and metastasis, as the authors listed [10]. Thus, A_2B_R antagonists are potentially a novel anticancer therapy, either in combination with other anticancer drugs or as a monotherapy. Indeed, several A_2B_R antagonists (i.e., AB928 26, PBF-1129 and theophylline 11) are now in clinical trials for patients with various types of cancers. Finally, Golay et al. performed a systematized survey and analysis of the literature to review the current status of animal and human research on G protein-coupled receptors (GPCRs) in the context of selected hematopoietic stem cell transplantation (HSCT) outcomes [12]. Interestingly, A_2A_R activation limits graft-versus-host disease after allogenic hematopoietic stem cell transplantation [13] and mediates an increase in donor-derived regulatory T cell suppression development of graft-versus-host disease [14].

Subsequently, nine research articles assess new functional, mechanistic, medicinal chemistry and pathophysiological prospects for ARs. Thus, Szabo et al. implemented the receptorial responsiveness method (RRM) to estimate the known concentrations of stable synthetic A_1_R agonists in isolated, paced guinea pig left atria [15]. Interestingly, the RRM is a procedure that is based on a simple nonlinear regression while using a model with two variables (X, Y) and (at least) one parameter to be determined (cx) [16]. Mocking et al. developed a bioluminescence resonance energy transfer (BRET)-based G protein-activation assay to probe duration of GPCR blockade [17]. Interestingly, the assay monitors heterotrimeric G protein activation via scavenging of released Venus-Gβ1γ2 by NanoLuc (Nluc)-tagged membrane-associated-C-terminal fragment of G protein-coupled receptor kinase 3 (masGRK3ct-Nluc) as a tool to probe duration of GPCR antagonism [17]. Next, Pelassa et al. provide biochemical (i.e., co-immunoprecipitation) and biophysical (i.e., proximity ligation assay) evidence confirming that endogenous A_2A_R and dopamine D_2_ receptor (i.e., D_2_R) heteromerize at the plasma membrane of rat striatal astrocytes [18]. Since striatal astrocytes are recognized to be involved in Parkinson’s disease (PD) pathophysiology, the findings reported here shed light on the molecular mechanisms involved in the pathogenesis of the disease. Borroto-Escuela et al. present further evidence that A_2A_R-D_2_R heteromers in the nucleus accumbens, through A_2A_R mediated allosteric inhibition of the D_2_R, can increase anti-reward in the ventral striatopallidal GABA neurons and inhibit cocaine self-administration, whereas the A_2A_R homodimer does not appear to be involved in this allosteric mechanism [19]. Subsequently, Fernández-Dueñas et al. describe the development of a new AlphaScreen assay to detect GPCR oligomers in post-mortem human brains, thus confirming for the first time the existence of A_2A_R/D_2_R heteromers in human caudate [20]. In brief, antibodies against A_2A_R and D_2_R were selectively labelled with donor and acceptor beads to engage in a singlet oxygen energy transfer, dependent on the formation of A_2A_R/D_2_R heteromers. Importantly, by using this approach, the authors show that the A_2A_R/D_2_R heteromerization status may be increased in the caudate from PD patients. Thus, restoring the unbalanced A_2A_R/D_2_R heteromer function potentially associated with PD may help to better understand the disease etiology and to design selective combined pharmacotherapeutic strategies [20]. Then, Okada et al. demonstrate that both acute and chronic administrations of therapeutic-relevant concentrations of carbamazepine (CBZ)—an anticonvulsive drug that also binds to adenosine receptors—suppress excitatory astroglial glutamatergic transmission associated with IP3-R and AMPA-R [21]. Importantly, the A_2A_R agonistic action of CBZ contributes to chronic mechanisms of carbamazepine against several neuropsychiatric disorders via inhibition of astroglial pathomechanisms of proinflammatory responses of IFNγ and TNFα [21]. Irrera et al. investigate the efficacy of polydeoxyribonucleotide (PDRN), a biologic A_2A_R agonist, in an experimental model of psoriasis-like dermatitis [22]. Indeed, PDRN decreased pro-inflammatory cytokines, prompted Wnt signaling, reduced IL-2 and increased IL-10. Thus, the authors concluded that PDRN anti-psoriasis potential may be linked to a “dual mode” of action: (i) NF-κB inhibition, and ii) Wnt/β-catenin stimulation [22]. Finally, Hayashi assesses the molecular and functional expression of adenosine receptors in the exocrine pancreases of rats, mice, and guinea pigs [23]. Interestingly, the author concludes that A_2A_R is a net contributor to exocrine secretion in the rodent pancreas, an assumption based on: (i) A_2A_R agonists stimulating a HCO_3_^−^-rich fluid secretion, and (ii) A_2A_R colocalizing with ezrin in the luminal membrane of duct cells [23]. Lertsuwan et al. propose a novel adenosine-mediated cancer cell growth and invasion suppression via a receptor-independent mechanism in cholangiocarcinoma (CCA) [24]. Indeed, the authors postulated a novel adenosine-mediated cancer cell suppression through a receptor-independent but nucleoside-transporter-dependent mechanism in CCA cells, thus extracellular adenosine treatment led to increased intracellular adenosine, which was later phosphorylated to 5′ AMP by adenosine kinase with the concomitant activation of 5′ AMP-activated protein kinase (AMPK) [24].

Overall, we hope that this timely focused issue summarizing our current knowledge on adenosine receptors will be of interest to a wide range of readers of the journal, interested in the purinergic field. Finally, we wish to express our best thanks to all authors and co-authors of the issue for their commitment and to the anonymous reviewers for their excellent contributions.
